# Repetitive Transcranial Magnetic Stimulation: A Potential Treatment for Obesity in Patients with Schizophrenia

**DOI:** 10.3390/bs11060086

**Published:** 2021-06-11

**Authors:** Ramey G. Monem, Olaoluwa O. Okusaga

**Affiliations:** 1 VA Pittsburgh Healthcare System, Pittsburgh, PA 15240, USA; ramey.monem@va.gov; 2 Bipolar and Schizophrenia Treatment (BeST), Repetitive Transcranial Magnetic Stimulation (rTMS) Clinic, Michael E. DeBakey VA Medical Center, Houston, TX 77030, USA; 3 Menninger Department of Psychiatry and Behavioral Sciences, Baylor College of Medicine, Houston, TX 77030, USA

**Keywords:** schizophrenia, obesity, brain, repetitive transcranial magnetic stimulation (rTMS), appetite, weight loss, neuroinflammation, brain reward circuitry, frontal cortex

## Abstract

Obesity is highly prevalent in patients with schizophrenia and, in association with metabolic syndrome, contributes to premature deaths of patients due to cardiovascular disease complications. Moreover, pharmacologic, and behavioral interventions have not stemmed the tide of obesity in schizophrenia. Therefore, novel effective interventions are urgently needed. Repetitive transcranial magnetic stimulation (rTMS) has shown efficacy for inducing weight loss in obese non-psychiatric samples but this promising intervention has not been evaluated as a weight loss intervention in patients with schizophrenia. In this narrative review, we describe three brain mechanisms (hypothalamic inflammation, dysregulated mesocorticolimbic reward system, and impaired prefrontal cortex function) implicated in the pathogenesis and pathophysiology of obesity and emphasize how the three mechanisms have also been implicated in the neurobiology of schizophrenia. We then argue that, based on the three overlapping brain mechanisms in obesity and schizophrenia, rTMS would be effective as a weight loss intervention in patients with schizophrenia and comorbid obesity. We end this review by describing how deep TMS, relative to conventional TMS, could potentially result in larger effect size for weight loss. While this review is mainly conceptual and based on an extrapolation of findings from non-schizophrenia samples, our aim is to stimulate research in the use of rTMS for weight loss in patients with schizophrenia.

## 1. Introduction 

The prevalence of obesity in patients with schizophrenia is as high as 60% [1] and the odds of obesity is thrice that of the general population [1]. Furthermore, the high prevalence of obesity in patients with schizophrenia is linked to a high rate of metabolic syndrome [2]. Obesity is also associated with cardiovascular disease (CVD) and patients with schizophrenia die from CVD complications 15–20 years earlier and at 2–3 times the rate in the general population [3]. While patients with schizophrenia might be genetically predisposed to obesity and other metabolic derangements (independent of psychiatric medications), psychotropic medications (especially certain antipsychotic medications) are a significant contributor to high obesity rates in schizophrenia [4]. 

So far, pharmacologic, and behavioral interventions have not been effective in stemming the tide of obesity in schizophrenia [5]. It is therefore important to identify novel interventions for the treatment and prevention of obesity in schizophrenia, which will ultimately reduce morbidity and premature death in this patient population. One such intervention is noninvasive neuromodulation—specifically, repetitive transcranial magnetic stimulation (rTMS)—a promising intervention for obesity [6,7]. While still in the preliminary phase, emerging data suggest that rTMS can reduce food craving and food consumption, and lead to weight loss in obese individuals [6,8,9]. However, the utility of rTMS for weight loss in obese patients with schizophrenia has not been evaluated. The unavailability of data on rTMS for treatment of obesity in patients with schizophrenia is related to the fact that patients with schizophrenia were excluded from the few controlled studies of rTMS for obesity. Therefore, as a first step in filling the void in the literature, we have carried out this narrative review to stimulate research in the field by highlighting the potential utility of rTMS for reducing food craving and food consumption, and inducing weight loss in obese patients with schizophrenia. 

In this review we will first discuss brain mechanisms implicated in obesity, emphasizing how these mechanisms have also been implicated in the neurobiology of schizophrenia. We will then describe the basics of rTMS, review the literature on the use of rTMS for treating obesity in non-schizophrenia samples, and explain why it is plausible to hypothesize that rTMS will be effective for reducing food cravings and excessive food consumption, promoting weight loss, and treating obesity in individuals with schizophrenia. In the final section, we will discuss deep TMS and why it is reasonable to speculate that, relative to conventional rTMS, deep TMS will have a larger effect size in the treatment of obesity in schizophrenia. 

## 2. The Brain and Obesity

The brain is involved in the control of appetite, energy intake, and expenditure [10]. The critical role of the brain in metabolic homeostatic control is reflected by the fact that obesity is now conceptualized as a brain disease [11]. While the hypothalamus acts as the key regulator of appetite and satiety (and hence, food consumption) [12], the mesolimbic reward system [13] and the prefrontal cortex [14] are also involved in the regulation of food consumption. Since weight gain occurs when energy intake exceeds expenditure, any impairment in the brain’s ability to optimally regulate energy intake will ultimately result in obesity. 

### 2.1. Brain Mechanisms of Appetite Regulation and Food Consumption

#### 2.1.1. Hypothalamic Appetite-Regulating System

The arcuate nucleus (ARC) of the hypothalamus contains two groups of neurons: (1) Neurons expressing proopiomelanocortin (POMC) and cocaine and amphetamine-regulated transcript (CART); (2) neurons expressing neuropeptide Y (NPY) and agouti-related protein (AgRP) [12]. POMC is a prohormone that is post-translationally cleaved into alpha-melanocyte-stimulating hormone (α-MSH), adrenocorticotropic hormone (ACTH), and beta-endorphin [15]. POMC/CART and NPY/AgRP neurons project to second order neurons in the paraventricular nucleus (PVN) of the hypothalamus [12]. Both the ARC and PVN neurons possess melanocortin receptors 3 and 4 (MC3R and MC4R); α-MSH is an agonist, while AgRP is an antagonist at both receptors [12]. PVN neurons project to the nucleus tractus solitarius (NST), an important brain region where sensing and integration of signals relevant for the control of feeding behavior occur [16]. 

POMC/CART neurons reduce appetite (i.e., they are anorexigenic) and promote increased energy expenditure, while NPY/AgRP neurons promote hunger (i.e., orexigenic) [17]. It is important to note that there is crosstalk between the brain, gut, and other peripheral metabolic organs (e.g., liver, pancreas, and adipose tissue) via neurohormones and sympathetic and parasympathetic innervation, respectively. The crosstalk between the brain and periphery is illustrated by the fact that food ingestion causes the secretion of peripheral signals of excess nutrients/energy stores such as leptin and insulin, which stimulate POMC/CART neurons while simultaneously inhibiting NPY/AgRP neurons [18]. As a result of the stimulation of POMC/CART neurons, α-MSH binds MC3R and MC4R receptors on the PVN neurons which in turn lead to satiety signals being sent to the NTS to ultimately reduce food intake and increase energy expenditure [12,16,19]. By contrast, fasting results in the production of hunger signals such as ghrelin, which stimulate NPY/AgRP neurons to increase food intake [12,16,19]. In summary, the POMC/CART and NPY/AgRP neurons function in a ‘Yin and Yang’ fashion to regulate food intake [17]. 

#### 2.1.2. The Mesocorticolimbic Reward System and Food Consumption

The reward circuitry in humans consists of dopaminergic neurons projecting from the ventral tegmental area (VTA) in the midbrain to the nucleus accumbens (NAc) (the mesolimbic pathway) and the prefrontal cortex (PFC) (the mesocortical pathway), respectively [20]. In addition to the primary projections to the NAc and PFC, VTA neurons also project to the cingulate cortex, hippocampus, amygdala, and olfactory tubercle [21]. The mesolimbic pathway to the NAc is involved in the positive reinforcing effects of natural rewards, drugs of abuse, as well as certain foods (described in the next paragraph below) [20], while the mesocortical pathway to the PFC is important for planning motivated behaviors (e.g., seeking and acquiring food, street drugs, and sex) and emotional responses [22]. Notably, the dopaminergic neurons in the VTA (which project to the NAc and PFC) possess receptors for hunger/satiation-mediated peptides including leptin, ghrelin, and orexin, findings that link the reward pathways to homeostatic processes of energy regulation [23].

Food ingestion can produce pleasurable, positively reinforcing, and rewarding effects. In fact, appetitive, hyperpalatable, calorie-dense, high-carbohydrate, high-fat foods are very salient, rewarding stimuli which (like drugs of abuse) can elicit the release of dopamine in the NAc which can reinforce consumption of these foods [14,24]. Therefore, food-related activation of the brain reward circuitry, the reinforcement of behaviors, and the production of memories can lead to food craving and consumption of larger amounts of food than was intended [14,24]. 

#### 2.1.3. The Prefrontal Cortex and Food Consumption

The most anterior part of the frontal cortex, i.e., the prefrontal cortex [14], contains the cortical networks that support behavioral regulation (i.e., the integration of cognitive processes including attention, working memory, and inhibitory control) [25]. Specifically, dietary self-regulation refers to an individual’s ability to exert conscious control over food choice and consumption [14]. As previously mentioned, certain foods provoke robust activation of neurons in the brain’s reward system (the VTA and NAc). Importantly, the dorsolateral prefrontal cortex (DLPFC) mediates appropriate cognitive strategies necessary to inhibit food-evoked visceral cravings (e.g., secondary to exposure to palatable food and food cues such as food advertisements), thus preventing overindulgence in the absence of physiological energy deficit/hunger [26]. In addition, the DLPFC implements cognitive control by modulating dopamine neurotransmission in the ventromedial PFC (VMPFC), VTA, and NAc, resulting in the inhibition of inappropriate responses, devaluation of immediate appetitive rewards, and the implementation of goal-directed behaviors [14]. In summary, the DLPFC is a critical functional node for the downregulation of the rewarding properties of energy-dense foods, inhibiting impulsive food consumption in the absence of a physiological energy deficit, thereby enabling individuals to exert control over their consumptive behaviors.

### 2.2. Brain Abnormalities Implicated in Dysregulation of Energy Homeostasis and Obesity

#### 2.2.1. Hypothalamic Inflammation and Obesity

Animal models and human studies have shown that inflammation of the hypothalamus leads to dysregulation of energy homeostasis, which subsequently results in obesity, glucose intolerance/diabetes, and hyperlipidemia [11,27]. Importantly, the observed hypothalamic inflammation is chronic, low-grade, nonsuppurative, and occurs without evidence of foreign substances in the brain. Imaging studies have also provided evidence in support of the existence of obesity-related hypothalamic neuroinflammation [28,29]. However, it is yet to be determined conclusively whether the hypothalamic inflammation seen in obese individuals is secondary to diet or whether it is independent of diet, and a part of the mechanism of disease [11,27]. Nevertheless, studies have shown that high-fat diet induces activation of glial cells (astrocytes and microglia), which then mount inflammatory responses which ultimately produce inflammation of the hypothalamus, specifically the POMC neurons in the ARC [11]. Moreover, obesity is associated with chronic low-grade peripheral inflammation [30], which can promote the migration of peripheral immune cells into the brain, further activating microglia, and contribute to hypothalamic inflammation [31]. In addition to activating glial cells, a high-fat diet also reduced the response of ARC neurons to exogenous leptin in mice [32] resulting in leptin resistance, a phenomenon associated with obesity [33]. While hypothalamic inflammation seems to perpetuate obesity, a limitation of the current studies on hypothalamic inflammation and obesity is that they do not clarify whether hypothalamic inflammation is a precursor (i.e., precedes weight gain) or strictly a consequence of overeating and obesity. 

#### 2.2.2. Dysregulated Mesocorticolimbic Reward System and Uncontrolled Food Consumption

Food ingestion (especially appetitive, hyperpalatable, calorie-dense foods) produces pleasurable and rewarding effects elicited by the release of dopamine in the NAc [24]. However, obesity maybe associated with blunting of the pleasurable and rewarding effects of food (the reward deficiency hypothesis) such that greater consumption of food might be required for an individual to experience the same levels of pleasure as non-obese individuals [24]. In the same vein, impaired dopamine receptor (D2/D3) binding in the ventral striatum was associated with obesity in some studies, suggesting that lower dopamine receptor binding probably led to excessive food consumption to compensate for blunted responses in the reward neural pathway [34,35]. However, prospective functional magnetic resonance imaging (fMRI) studies did not confirm an association between dopamine occupancy in the ventral striatum and the risk of obesity in non-psychiatric samples [36]. 

Another construct which is biologically mediated by the mesocorticolimbic reward system is food reward sensitivity (the tendency to seek and derive pleasure from food), which has also been positively correlated with food addiction, uncontrolled eating, and obesity [37,38]. Additionally, impaired mesocorticolimbic reward circuitry may predispose an individual to the overconsumption of hyperpalatable calorie-dense foods due to an increase in the reward values assigned to such foods [36]. In conclusion, dysregulated mesocorticolimbic reward function may be associated with uncontrolled food intake and obesity, and this association could be related to impaired dopaminergic transmission in the ventral striatum, but well-designed prospective studies are needed to clarify the role of mesocorticolimbic dopamine neurotransmission in the pathogenesis and perpetuation of obesity. 

#### 2.2.3. Impaired Prefrontal Cortex Function and Food Consumption

Neuroimaging studies have demonstrated a negative relationship between body mass index and DLPFC activation, suggesting that individuals with obesity may have greater difficulty utilizing the DLPFC when making food consumption decisions [39,40]. In addition, experimentally induced inhibition of the left DLPFC using noninvasive brain stimulation techniques resulted in increased appetitive snack food consumption [41,42]. Importantly, the participants in the DLPFC inhibition study were of normal weight, which suggests that impaired DLPFC function precedes the onset of obesity and could have a causal role in obesity [41,42]. In addition, obese individuals have been shown to exhibit reduced grey matter volume in the DLPFC when compared with lean individuals [43]. The DLPFC is also a prominent brain region involved in cognitive control, one hallmark of which is the ability to easily disengage from one train of thought or activity to seek an alternative [44]. Therefore, people with impaired DLPFC function may find it difficult to disengage from food cravings and ultimately end up overconsuming food. Finally, impaired DLPFC function is associated with impulsivity, which (along with poor cognitive inhibitory control) is associated with delay discounting (a tendency to choose small, immediate rewards over larger, delayed rewards) [45]; impulsivity and delay discounting have been associated with overconsumption of highly palatable foods [46]. 

### 2.3. Brain Abnormalities Implicated in Dysregulated Energy Homeostasis and Obesity Have Also Been Implicated in the Neurobiology of Schizophrenia

**Figure 1:**
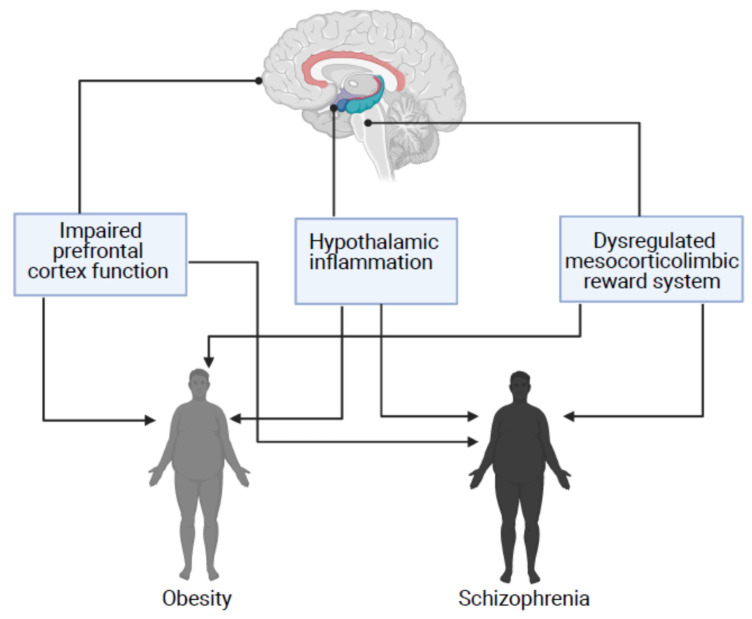
Three overlapping brain mechanisms in obesity and schizophrenia. Impaired prefrontal cortex function, hypothalamic inflammation and dysregulated mesocorticolimbic reward system have been implicated in the pathogenesis of obesity and have also been described as part of the neurobiology of schizophrenia.

Interestingly, the previously described brain abnormalities linked to impaired energy homeostasis and obesity have been described as part of the neurobiology of schizophrenia, which suggests that patients with schizophrenia might have an underlying vulnerability to excessive food consumption and obesity (Figure 1).

#### 2.3.1. Hypothalamic Inflammation in Schizophrenia

Immune activation and inflammation have been found in the periphery and in the brain of patients with schizophrenia [47,48]. The chronic low-grade inflammation in the brain (neuroinflammation) of patients with schizophrenia is thought to be related to microglial activation [49]. However, neuroinflammation might be relevant for only a subset of patients and not across the board [50], an idea consistent with the heterogeneity found in the illness [51]. Of note, post-mortem examination revealed chronic low-grade inflammation of the hypothalamus in patients with schizophrenia [52], although there was insufficient information to specifically link the inflamed hypothalamus to obesity in the patients. Additionally, atypical antipsychotics (now the mainstay of treatment of schizophrenia) have been causally linked to hypothalamic inflammation in animal models (in vitro and in vivo) [53,54,55] and it is therefore reasonable to speculate that hypothalamic inflammation in patients with schizophrenia might be linked to treatment with atypical antipsychotic medications. Moreover, the two atypical antipsychotic medications with the highest liability for increasing appetite (clozapine and olanzapine) and inducing obesity in patients are also the ones with the strongest evidence for inducing hypothalamic inflammation in animal models [53,54,55]. 

#### 2.3.2. Dysregulated Mesocorticolimbic Reward System in Patients with Schizophrenia

Dopamine neurotransmission plays a critical role in reward processing [20] and is also the neurotransmitter system consistently implicated in the neurobiology of schizophrenia [56]. Moreover, all antipsychotic medications currently approved for schizophrenia target dopamine. Specifically, abnormal mesolimbic and mesocortical brain connectivity involving dopamine neurotransmission are longstanding models posited to explain positive (e.g., delusions and hallucinations) and cognitive/negative (e.g., executive function deficits, amotivation, and alogia) symptoms of schizophrenia [56]. Considering the role of the mesocorticolimbic reward system in controlling food intake (please refer to the earlier sections of this review) [20,22,23], abnormalities of the reward circuitry may predispose patients with schizophrenia to abnormal eating behaviors that can lead to obesity. Interestingly, patients with schizophrenia and comorbid metabolic syndrome (truncal obesity, hypertension, dyslipidemia, and glucose intolerance) were found to have smaller reward-related brain structures than patients without metabolic syndrome [57], a finding that supports a role of the brain reward circuitry in the development of obesity in patients with schizophrenia. 

Moreover, blockade of dopamine receptors in the mesocorticolimbic neural pathway by antipsychotic medications could also contribute to impairments that result in unhealthy eating behaviors in patients with schizophrenia. Indeed, it has been posited that blockade of dopamine receptors in the nucleus accumbens by antipsychotics could reduce the pleasurable and rewarding effects of food, causing patients to compensate by increasing their food intake, especially calorie-dense, hyperpalatable food [58,59]. In this vein, studies have shown that, relative to second-generation antipsychotics, first-generation antipsychotics (which have stronger dopamine-blocking activities) result in less activation of the ventral striatum using a monetary incentive delay task (MID) [60]. While MID is not a food-related reward paradigm, it is a validated measure of reward function. However, brain reward abnormalities might be intrinsic to schizophrenia, as unmedicated patients with schizophrenia [61,62] and their healthy first-degree relatives [63] exhibited reduced activation of the ventral striatum during reward anticipation. Finally, the magnitude of ventral striatal activation during reward anticipation negatively correlated with antipsychotic-induced weight gain in patients with schizophrenia such that those with the least striatal reward activity at baseline, gained the most weight after six weeks of antipsychotic treatment [64]. 

#### 2.3.3. Impaired Prefrontal Cortex (PFC) Function in Patients with Schizophrenia

Individuals with schizophrenia have been shown to exhibit structural and functional impairments in their PFC [65]. PFC abnormalities in patients with schizophrenia are related to cognitive function (working memory and other executive functioning) deficits. Specifically, neuroimaging studies have shown that the dorsolateral prefrontal cortex (DLPFC) of patients with schizophrenia function less efficiently in comparison with healthy controls [66,67], similar to reports of dysfunction in the same brain region of individuals with obesity [14]. In addition, dysfunction in the PFC is linked to poor decision-making and impulsivity in individuals with schizophrenia [68,69]. Poor decision-making and lower levels of inhibitory control secondary to impaired DLPFC function in patients could then extend to decision-making about food such that patients excessively consume unhealthy foods that are likely to result in obesity [58]. 

Although we have a made a distinction between the prefrontal cortex (cognitive function) and reward circuitry, emerging data suggest a dysfunctional interaction between prefrontal cortex and reward circuits in patients with schizophrenia [70]. For example, patients with schizophrenia exhibit delayed discounting deficits, i.e., relative to healthy controls, they will choose a significantly smaller immediate reward over a larger delayed reward [71] which might be related to prefrontal cortex-related inability to represent the value of outcomes and plans [70,72]. Importantly, cognitive function, specifically episodic and working memory, inversely correlated with the degree of discounting of the value of future rewards in patients [72]. It is therefore possible that patients with schizophrenia choose appetitive, hyperpalatable, calorie-dense, high-carbohydrate, high-fat foods over healthier foods because of the immediate hedonic response (i.e., immediate reward) of appetitive, unhealthy foods but attach less value to the larger, delayed health rewards of healthy foods. 

## 3. Repetitive Transcranial Magnetic Stimulation (rTMS) as a Potential Treatment for Obesity in Schizophrenia

### 3.1. The Basics of rTMS

From a historical perspective, the prototype of the modern day transcranial magnetic stimulation (TMS) machine was designed by Barker and his colleagues in 1985 at the University of Sheffield, England [73,74]. Transcranial magnetic stimulation (TMS) involves the use of an alternating magnetic field to induce electric current in cortical brain tissue using a coil placed on the scalp [73]. TMS is based on the principle of induction of a magnetic field by an alternating electric current flowing through a coil (ampere’s law), and the induction of an electric current by an alternating magnetic field (Faraday’s law) [74]. The electric current induced in the cortical tissue by the magnetic field emanating from the TMS coil will cause the depolarization of neurons which will produce an action potential and firing of neurons which will subsequently have behavioral effects. In general, the magnetic fields generated by commercially available figure-of-eight (figure-8 coils) TMS coils, which is on average 1.5 to 4 Tesla, do not extend into the brain beyond 2.5 cm from the scalp, although H-coils penetrate deeper to levels up to 6 cm [75]. TMS can be delivered as single pulses or in a repeated rhythmic fashion referred to as repetitive TMS (rTMS) [76]. Moreover, the frequency of the pulses in rTMS is said to be “low” if ≤ 1 Hz or “high” if ≥ 1 Hz [76]. A single pulse of TMS can result in quick, immediate behavioral changes, such as involuntary movement or perceived flashes of light, depending on the area of the brain being targeted [77]. However, rTMS results in changes in neuronal activity and brain function that last well beyond the initial stimulation and is used to induce longer-lasting changes in behavior and cognitive processing [73]. In psychiatry, rTMS (usually high frequency) is approved for treatment-resistant major depressive disorder [78]. Deep TMS, which uses the H-coil, has been approved for obsessive–compulsive disorder [79]. 

For the treatment of major depressive disorder, the procedure for most protocols involves daily treatment sessions (usually Monday to Friday) lasting 20–30 min for a total of 30 treatment sessions with the TMS coil placed on the left DLPFC [78]. The commonest side effect of rTMS is local scalp/facial pain or headache, related to the stimulation of peripheral scalp/facial muscles at the point of contact of the TMS coil; the pain or headache can usually be treated with acetaminophen or aspirin [74]. The continuous clicking sound of the TMS machine could potentially affect a person’s auditory threshold and for this reason, it is standard practice for patients and treaters to wear earplugs during treatment sessions [78]. A rare but more serious side effect of rTMS is seizure, which is self-limiting and does not result in epilepsy (i.e., seizure disorder) [74,78]. The risk of seizure increases with higher stimulaton intensity [74]. Since rTMS involves the application of a magnetic field to the brain, it is contraindicated in individuals with ferromagnetic implants [78]. 

### 3.2. Efficacy of rTMS for Reducing Food Craving, Food Consumption and Treating Obesity in Non-Psychiatric Samples

The awareness of the DLPFC’s role in dysregulated eating behaviors (discussed in earlier sections of this review), has resulted in increased interest in rTMS targeting the DLPFC for modulating eating behaviors, reducing food cravings, and secondarily treat obesity [80,81]. Of note, rTMS applied to the DLPFC of smokers and cocaine users reduced subjective craving for cigarettes and cocaine [82], and based on the similar appetitive qualities of both substance use disorders to those of food in individuals with dysregulated eating, suggest that the same methods could be applied to control food cravings. Indeed, meta-analyses have shown that single-, and multi-session rTMS reduce food craving and food consumption with multi-session rTMS being associated with larger effect sizes [7]. While there is good evidence supporting the efficacy of rTMS in reducing food craving and consumption, there is a relative paucity of studies specifically evaluating the efficacy of rTMS for weight loss in obese individuals. To our knowledge, only three randomized, sham-controlled studies of high frequency rTMS [6,9,83] and one study of deep TMS [8] are currently available and all four studies resulted in significant weight loss in the active treatment group relative to the sham treatment group (Table 1). 

## 4. rTMS Effects That We Hypothesize to Be Relevant for Reducing Food Craving, Food Consumption and Inducing Weight Loss in Obese Patients with Schizophrenia 

**Figure 2:**
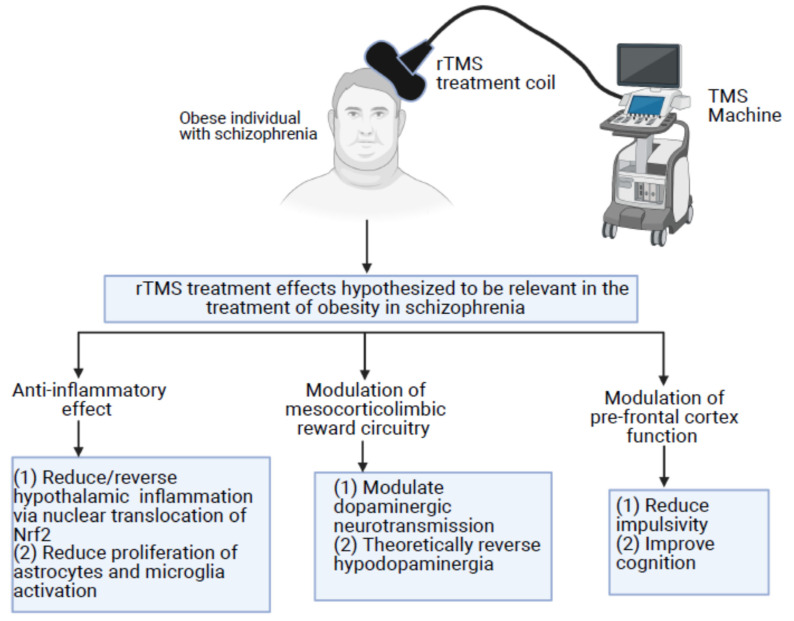
rTMS effects that we have hypothesized to be relevant for treating obesity in patients with schizophrenia. The documented effects of rTMS, including its anti-inflammatory effects, modulation of dopaminergic neurotransmission in the mesocorticolimbic system, and modulation of prefrontal cortex function, all have the potential to be beneficial for reducing food cravings and food consumption and lead to weight loss in patients with schizophrenia comorbid with obesity. rTMS: Repetitive transcranial magnetic stimulation; Nrf2: Nuclear factor erythroid-2-related factor 2.

We hypothesize that rTMS will reduce food cravings, which will secondarily lead to a reduction in calorie intake, and ultimately result in weight loss in obese patients with schizophrenia via three mechanisms, namely: (1) anti-inflammatory effect, (2) modulation of mesocorticolimbic reward circuitry, and (3) modulation of pre-frontal cortex function (Figure 2). 

### 4.1. Anti-Inflammatory Effect of rTMS

rTMS could potentially be effective for treating obesity in schizophrenia by reducing hypothalamic inflammation associated with schizophrenia [52] and implicated in the pathogenesis of obesity [11,27]. In vitro animal and human studies have demonstrated the anti-inflammatory effects of rTMS in the periphery and in the brain [84,85,86,87,88]. Pre-clinical studies indicate that the anti-inflammatory effect of rTMS is mediated by increased translocation of the transcription factor, nuclear factor erythroid-2-related factor 2 (Nrf2) into the nucleus of brain cells [89,90]. Interestingly, ziprasidone (an antipsychotic medication approved for the treatment of schizophrenia and also considered to possess the least risk of weight gain [91]) also increased the translocation of Nrf2 from the cytoplasm to the nucleus [92], suggesting that this shared mechanism with rTMS could be responsible for its relatively low liability for weight gain in patients. Theoretically, rTMS could also potentially be anti-inflammatory via its effect on glial cells, since high frequency rTMS was shown to inhibit the proliferation of astrocytes and reduced microglial activation in rodent CNS [93,94]; reactive astrocytes and activated microglia can contribute to neuroinflammation and neuronal death [95] and have been implicated in the pathophysiology of schizophrenia [96].

### 4.2. Modulation of the Mesocorticolimbic Reward Circuitry by rTMS

rTMS targeting the frontal cortex has a local effect on the neurons directly under the TMS coil but can also modulate subcortical networks, including the striatum, and hence influence the mesocorticolimbic reward network. In rodents, high frequency rTMS applied to the frontal cortex, resulted in increased dopaminergic neurotransmission in the mesocorticolimbic tract [97]. Similarly, in human subjects without schizophrenia, TMS applied to the anterior frontal cortex modulated reward-related processing in the striatum [98], and high-frequency rTMS to the DLPFC increased dopamine neurotransmission in the striatum [99,100]. Therefore, considering the hypothesized blunted reward response secondary to hypodopaminergia in the reward circuitry of patients with schizophrenia [64], we argue that rTMS applied to the DLPFC of obese individuals with schizophrenia could ameliorate the hypodopaminergia and reduce excessive food consumption and subsequently result in weight loss. Furthermore, it is plausible to speculate that the enhancement of dopamine neurotransmission in the striatum by rTMS could act in a similar manner as stimulants, which also enhance dopamine neurotransmission in the striatum and decrease appetite, and consequently weight loss [101]. However, the potential to worsen psychosis secondary to increased dopamine neurotransmission in the striatum [102], must be taken into consideration when planning to administer rTMS for obesity in patients with schizophrenia. The risk of worsening psychotic symptoms notwithstanding, it is important to note that high frequency rTMS applied to the left DLPFC has also been found to reduce psychotic symptoms in patients with schizophrenia [103]. 

### 4.3. Modulation of Pre-Frontal Cortex Function by rTMS 

We also hypothesize that rTMS applied to the left DLPFC of obese individuals with schizophrenia would result in reduced food craving, consumption and ultimately, weight loss, via rTMS modulatory effect on the pre-frontal cortex. Specifically, rTMS could reduce impulsivity, and translate to reduced impulsive and compulsive eating in obese patients with schizophrenia. For example, in patients with cocaine use disorder who also exhibit impulsive behavior, rTMS applied to the left DLPFC increased the functional connectivity between the left DLPFC and ventromedial prefrontal cortex and reduced impulsivity in the patients [104]. rTMS applied to the left prefrontal cortex also reduced behavioral impulsivity in patients with methamphetamine use disorder [105]. In addition, bilateral high frequency rTMS applied to the DLPFC reduced craving for cigarettes in patients with schizophrenia, a finding that further highlights the need to test the efficacy of rTMS for reducing food craving in patients with schizophrenia. Moreover, since cognitive function inversely correlated with the degree of discounting of the value of future rewards in patients with schizophrenia, rTMS might also reduce food consumption by improving overall cognitive function; of note, adjunctive high frequency rTMS applied to the left DLPFC of patients with schizophrenia improved multiple domains of cognition in the patients [103]. In summary, based on similarities between substance use disorders and obesity, we argue that the observed efficacy of rTMS in reducing impulsivity and craving in substance use disorders is likely to be replicable in obese patients with schizophrenia and the cognitive-enhancing properties of rTMS in patients with schizophrenia is also likely to result in healthier food consumption. 

## 5. Deep TMS

Unlike conventional TMS (with Figure-8 coils), which does not modulate cortical excitability beyond a depth of 2.5 cm from the scalp, deep TMS (dTMS) with H-coil can modulate cortical excitability up to a maximum depth of 6 cm and is therefore preferred for modulating the activity of deeper neuronal circuits [75,106]. Moreover, conventional TMS misses the cortical target in 27–32% of patients if neuronavigation is not used but dTMS is unlikely to miss the target because it stimulates larger areas of brain tissue (approximately 17 cm^3^ for dTMS vs. 3 cm^3^ for conventional TMS) [107]. Therefore, based on the properties of dTMS, it is reasonable to postulate that dTMS would have a larger effect size when used for the treatment of obesity in patients with schizophrenia. Consistent with our hypothesis of greater effect size of obesity treatment with dTMS vs. conventional TMS, the only available study (to our knowledge) of dTMS for weight loss in a non-psychiatric sample [8] revealed a larger decrease in weight (−7.83 ± 2.28 kg) when compared to weight loss in the three studies [6,9,83] that used conventional TMS (−1.35 ± 2.31 kg, −2.75 ± 2.37 kg, and −1.31 ± 1.3 kg, respectively)

## 6. Concluding Remarks and Future Directions

Based on certain shared neurobiological mechanisms between obesity and schizophrenia and data (although limited) showing the effectiveness of rTMS for obesity in non-psychiatric samples, we propose that rTMS is a potential treatment for obesity in patients with schizophrenia. However, there is currently no data on the effectiveness of rTMS for reducing food craving and consumption and inducing weight loss in obese individuals with schizophrenia. There is therefore an urgent need for clinical trials of rTMS for obesity in patients with schizophrenia, considering the epidemic of obesity in this patient population. Since our goal is to stimulate research on rTMS for obesity in patients with schizophrenia, a list of some of the questions that must be answered by future studies include: (1) What would be the optimal stimulation parameters? (2) Should rTMS be administered only to the left side, only to the right side, or bilaterally to the brain? (3) What would be the optimal duration of treatment? (4) What safety issues would arise? (5) Would rTMS in combination with another obesity intervention be superior to rTMS alone? (6) What would be the specific neurobiological substrates of the potential clinical benefits of rTMS for obesity in patients with schizophrenia?

## Figures and Tables

**Table 1:** Randomized sham-controlled studies of rTMS for weight loss in non-psychiatric samples.

Study	TMS Modality	Study Design	Inclusion Criteria	Treatment Allocation	Results
Kim et al. 2018 [6].	HF rTMS (10Hz) to left DLPFC, 4 sessions/week for 2 weeks vs. sham TMS.	Randomized, sham-controlled, single-blind, parallel-group trial.	Male or female, between 18 and 65 years, BMI ≥ 25, no psychiatric illness	30 randomized to active treatment and 30 to sham stimulation	Greater weight loss from baseline for active vs. sham stimulation (−1.35 ± 2.31 kg vs. 0.45 ± 1.28 kg; *p* = 0.002)
Kim et al. 2019 [9].	HF rTMS (10Hz) to left DLPFC, 8 sessions/week for 4 weeks vs. sham TMS.	Randomized, sham-controlled, single-blind, parallel-group trial.	Male or female, between 18 and 70 years, BMI ≥ 25, no psychiatric illness	21 randomized to active treatment and 22 to sham stimulation	Greater weight loss from baseline for active vs. sham stimulation (−2.75 ± 2.37 kg vs. 0.38 ± 1.0 kg; *p* < 0.01)
Ferrulli et al. 2019 [8].	HF dTMS (18 Hz) vs. LF dTMS (1 Hz) or sham.	Randomized, sham-controlled, single-blind, parallel-group trial.	Male or female, between 22 and 65 years, BMI between 30–45, no psychiatric illness	15 randomized to HF dTMS, 12 to LF dTMS and 12 to sham stimulation.	significant decrease in weight (−7.83 ± 2.28 kg; *p* = 0.0009) and BMI (−2.83 ± 0.83, *p* = 0.0009) for HF dTMS group.
Encarnacion et al. 2020 [83].	HF rTMS (10Hz) to left DLPFC, 2 sessions/week for 2 weeks vs. sham TMS.	Randomized, sham-controlled, single-blind, parallel group trial,	Male or female, between 15 and 65 years, BMI ≥ 30, no psychiatric illness	15 randomized to active treatment and 15 to sham stimulation	significant decrease in weight (−1.3 ± 1.3 kg; *p* =0.009) and BMI (0.6 ± 0.6, *p* =0.001) in the HF rTMS group.

HF = high frequency. LF = low frequency. DLPFC = dorsolateral prefrontal cortex. BMI = body mass index. rTMS = repetitive transcranial magnetic stimulation. dTMS = deep transcranial magnetic stimulation. Sham treatment is administered by placing the coil away from the treatment target (DLPFC) and stimulating at subtherapeutic intensity.

## Data Availability

Not applicable.
